# Supplementary figures and images for: Pseudomonas aeruginosa senses and responds to epithelial potassium flux via Kdp operon to promote biofilm
Source: PLoS Pathog. 2024 May 31;20(5):e1011453. doi: 10.1371/journal.ppat.1011453 (PMC11168685; doi:10.1371/journal.ppat.1011453)

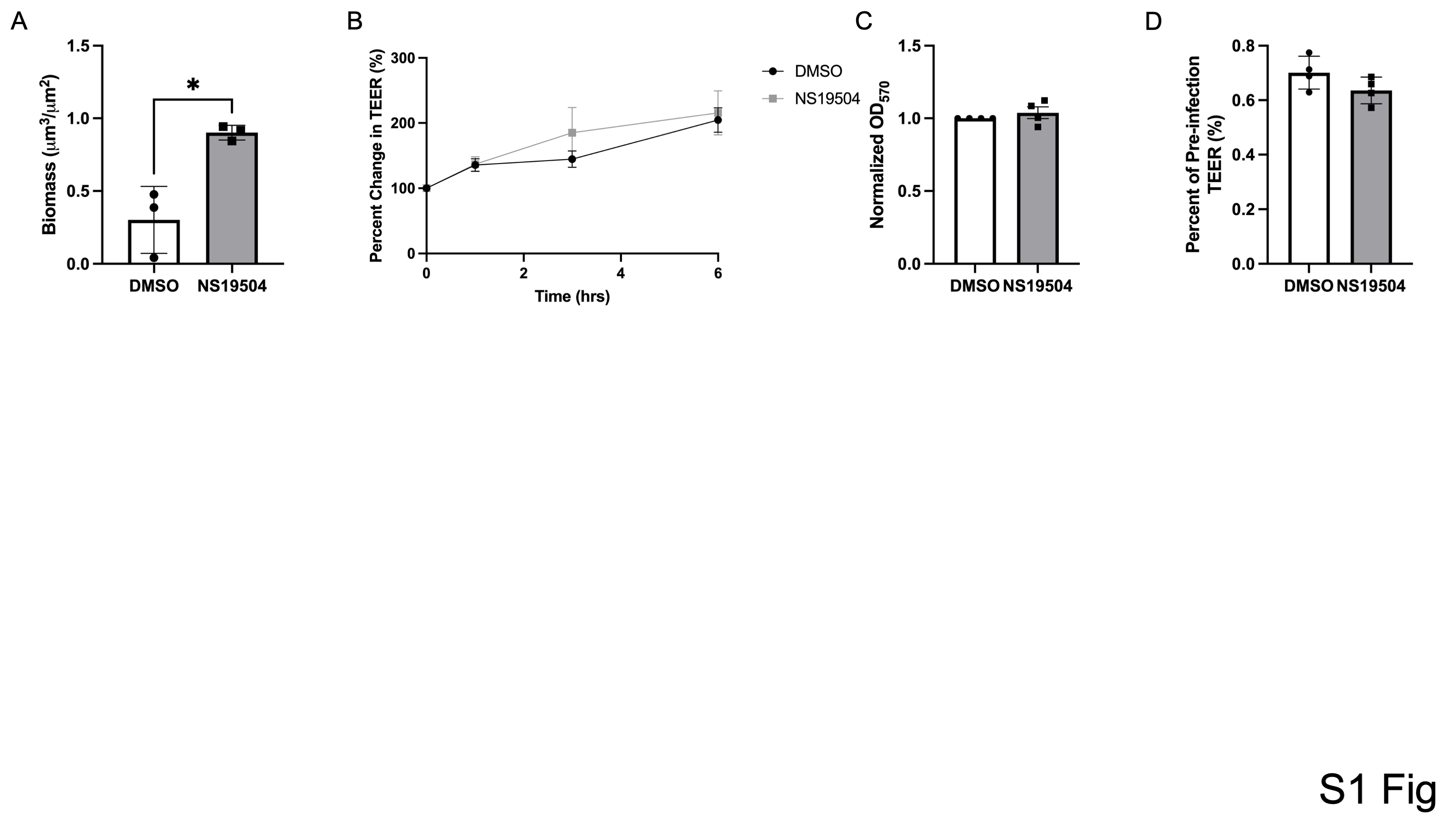

Supplement: S1 Fig — (A) CFBE41o- cells on glass coverslips were infected with GFP-producing P. aeruginosa (green) while stimulated with NS19504 (25 μM), a BKCa channel potentiator, or 0.05% DMSO in MEM without phenol red and imaged by fluorescent microscopy at 12-hours. Biomass (μm3/μm2) was measured at 12-hours post-inoculation from three independent experiments. Line in bar represents mean value and error bars represent standard error of the mean. Statistical significance was tested by unpaired t-test (* p<0.05). (B) Percent change in transepithelial resistance (TEER) measurements of differentiated CFBE41o- cells grown at air liquid interface after treatment with 25 nM NS19504 or 0.05% DMSO for 1, 3, and 6 hours. (C) Percent MTT assay absorbance compared to DMSO treated cells for NS19504 treated CFBE41o- cells grown after 6 hours of infection with P. aeruginosa. (D) Percent change in TEER for CFBE41o- cells after 6 hours of infection with P. aeruginosa while cells were treated with DMSO or NS19504. (TIFF) [file ppat.1011453.s001.tiff]

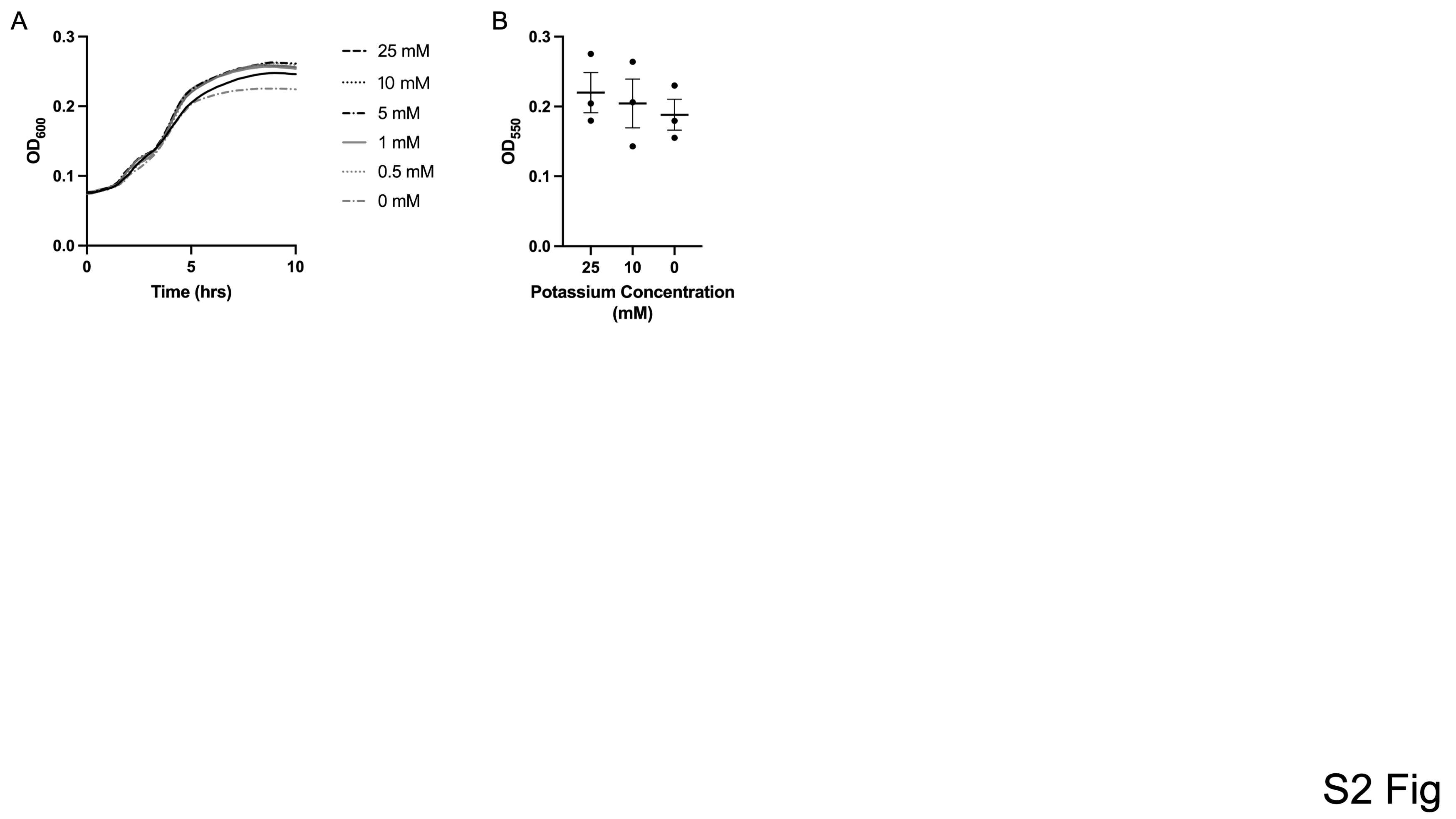

Supplement: S2 Fig — (A) Planktonic growth curves of PAO1 grown in M9 media without potassium or supplemented with 0.5, 1, 5, 10, and 25 mM potassium chloride showing no difference in planktonic growth. (B) P. aeruginosa biofilms grown in 96-well plates in M9 without potassium or supplemented with 10 or 25 mM potassium chloride measured using crystal violet absorbance at 550 nm. (TIFF) [file ppat.1011453.s002.tiff]

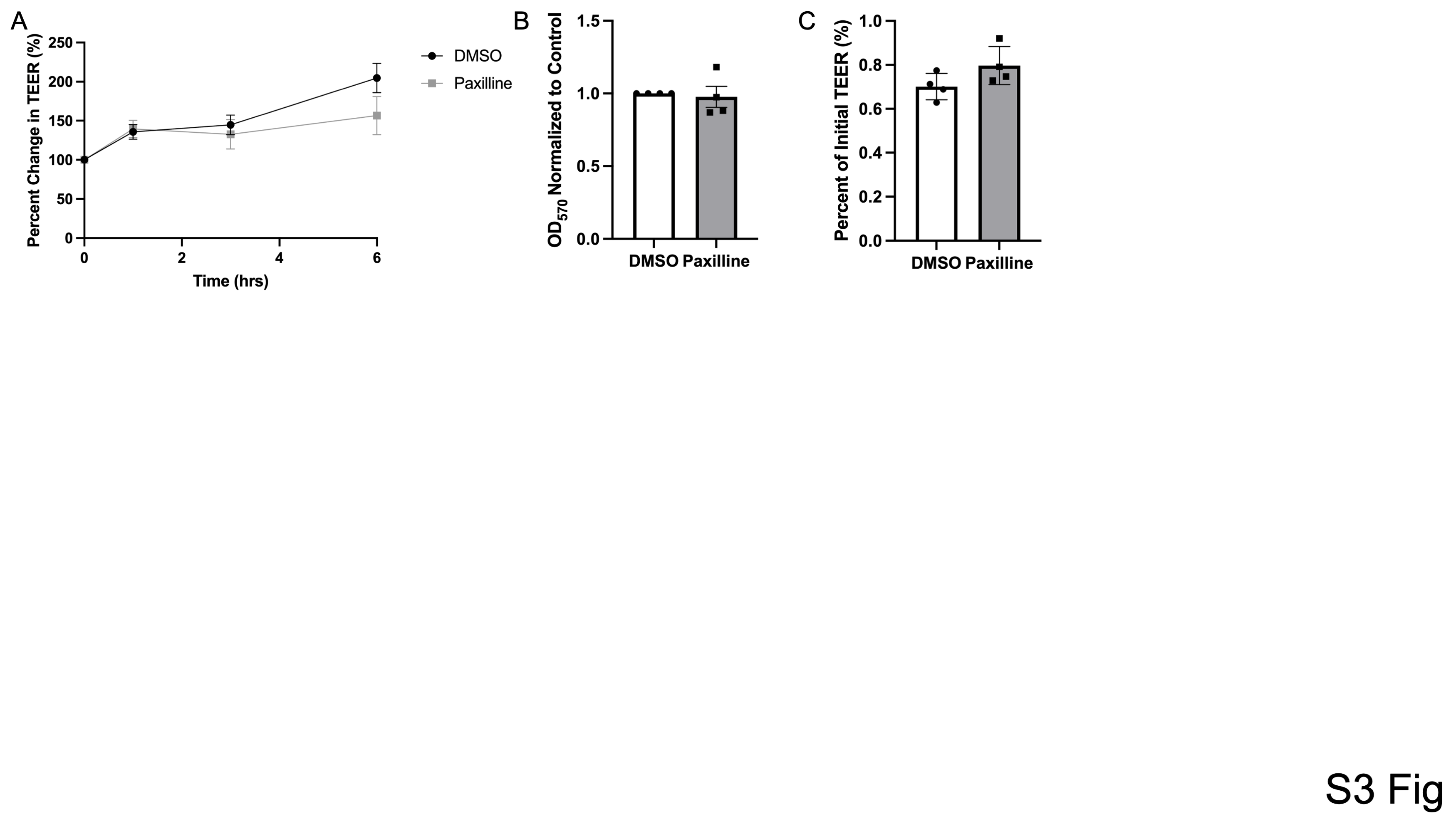

Supplement: S3 Fig — (A) Percent change in transepithelial resistance (TEER) measurements of differentiated CFBE41o- cells grown at air liquid interface after treatment with 10 nM paxilline or 0.05% DMSO for 1, 3, and 6 hours. (B) Percent MTT assay absorbance compared to DMSO treated cells for paxilline treated CFBE41o- cells grown after 6 hours of infection with P. aeruginosa. (C) Percent change in TEER for CFBE41o- cells after 6 hours of infection with DMSO or paxilline. (TIFF) [file ppat.1011453.s003.tiff]

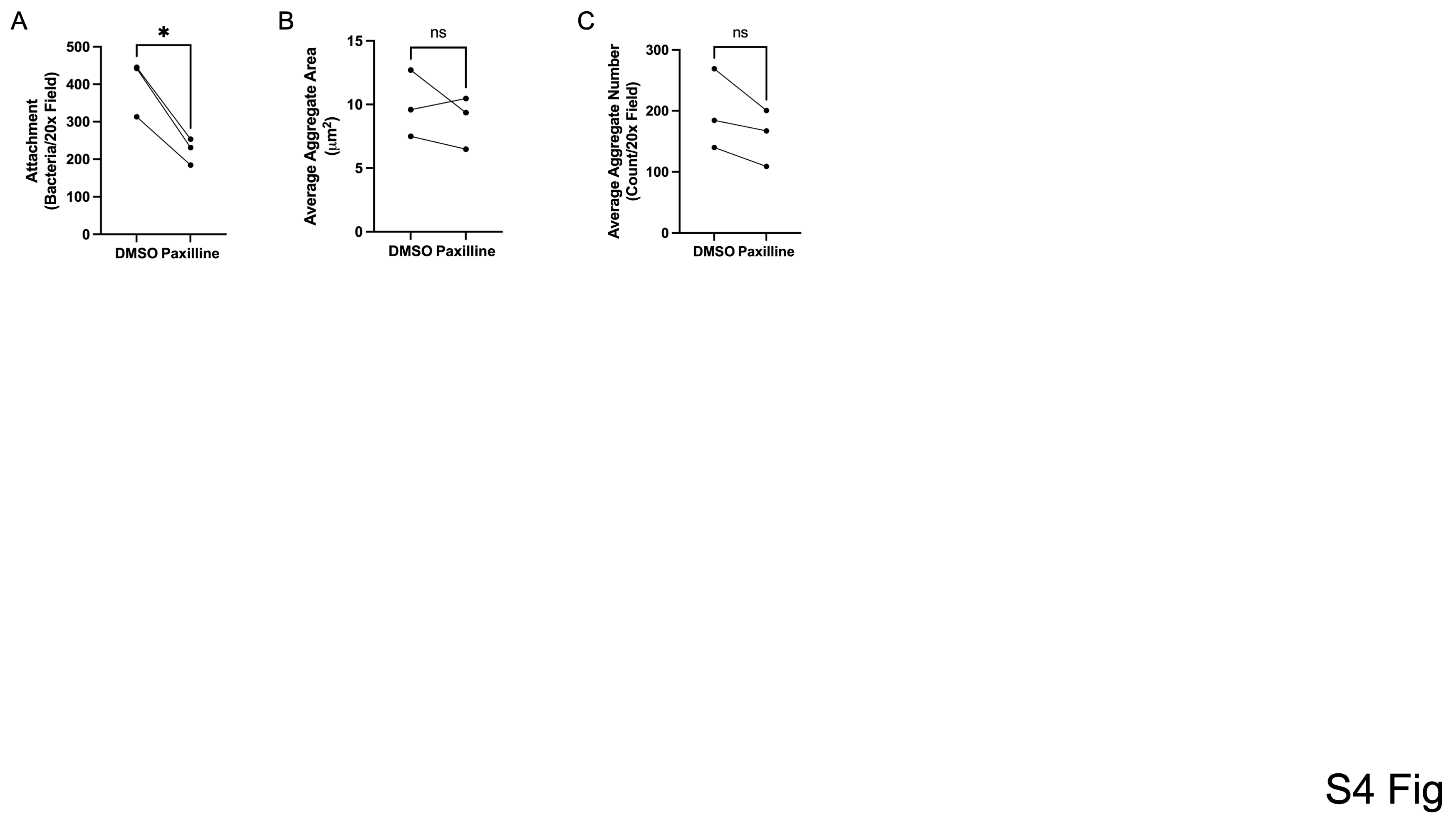

Supplement: S4 Fig — (A-C) Bacterial attachment at 1 hour and average aggregate area and number at 6 hours grown on CFBE41o- cells in live-cell co-culture experiments were measured using Nikon Elements. (A) Number of bacteria attached per 20x field for epithelial cells treated with 0.05% DMSO or paxilline (10 μM) during live-cell co-culture experiments at 1 hour. (B) Average aggregate number per 20x field measured at 6-hour time point for epithelial cells treated with 0.05% DMSO or paxilline (10 μM) during live-cell co-culture experiments. (C) Average aggregate area per 20x field measured at 6-hour time point for epithelial cells treated with 0.05% DMSO or paxilline (10 μM) during live-cell co-culture experiments. Line connecting data points indicates data points from say biologic replicate. Statistical significance was tested by unpaired t-test (* p<0.05). (TIFF) [file ppat.1011453.s004.tiff]

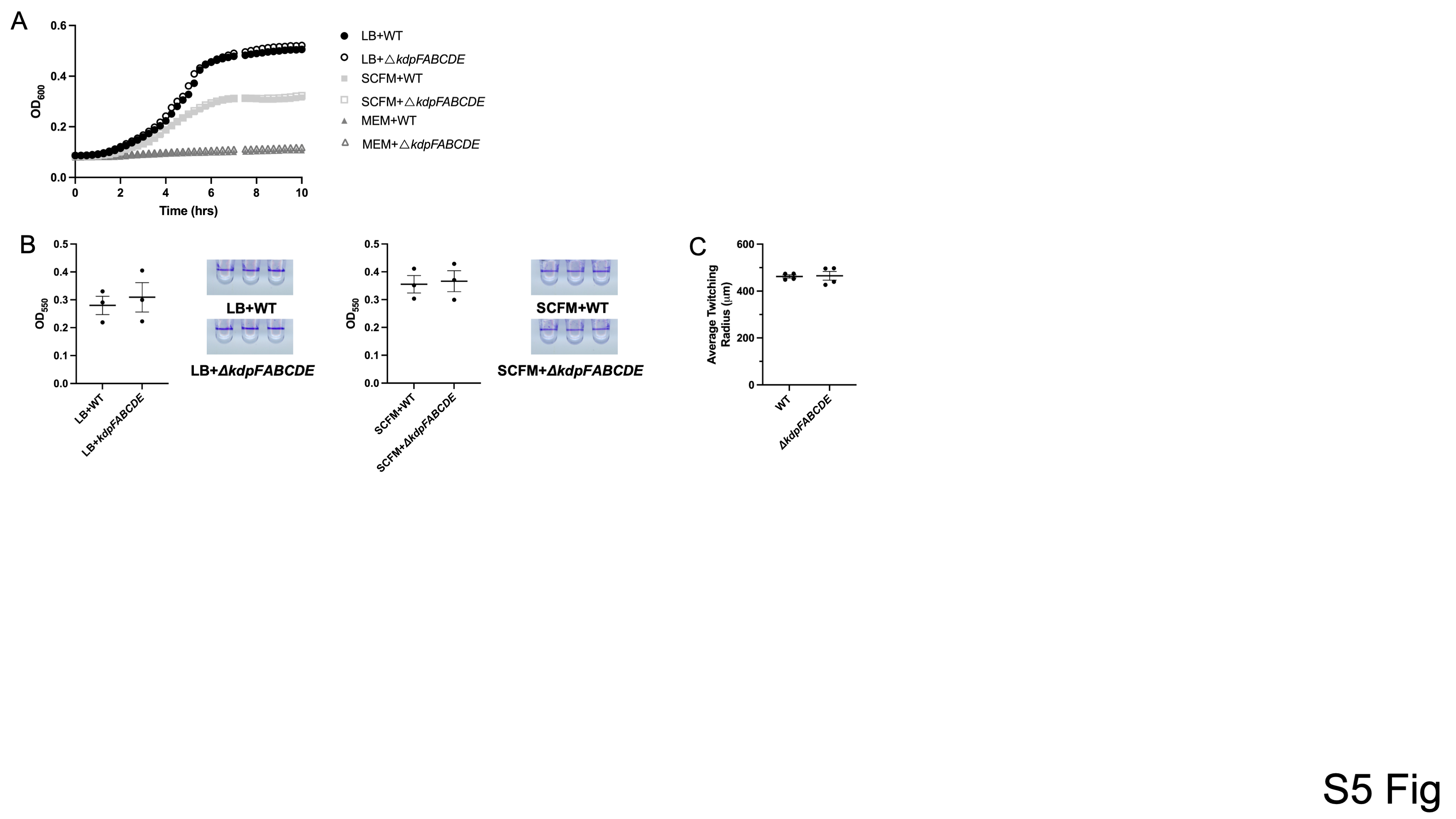

Supplement: S5 Fig — (A) Planktonic growth kinetics of WT PAO1 strain and ΔkdpFABCDE PAO1 strain grown in LB Lennox broth (LB), minimal essential media (MEM), and synthetic cystic fibrosis sputum media (SCFM). (B) Biofilm growth of WT PAO1 strain and ΔkdpFABCDE PAO1 strain in 96-well plates in LB and SCFM with or without NS19504 measured using crystal violet absorbance at 550 nm. Line represents mean and error bars represent standard error of the mean. (C) Average twitching motility radius of WT PAO1 strain and ΔkdpFABCDE PAO1 strain grown under M9 media supplemented with 1.5% agar. Line represents mean and error bars represent standard error of the mean. (TIFF) [file ppat.1011453.s005.tiff]
