# Supplementary material for: Pseudomonas aeruginosa senses and responds to epithelial potassium flux via Kdp operon to promote biofilm
Source: PLoS Pathog. 2024 May 31;20(5):e1011453. doi: 10.1371/journal.ppat.1011453 (PMC11168685; doi:10.1371/journal.ppat.1011453)
Supplement: S1 Table — (DOCX) [file ppat.1011453.s006.docx]

**S1 Table**

| **Strain** | **Insertion/Plasmids** | **Source** |
| --- | --- | --- |
| PAO1 | Wild type strain | Laboratory bacterial strain provided by George O’Toole Lab |
| PAO1:GFP | pSMC21 | [1-3] |
| PAO1:mTFP1 | pUC18-miniTn7T2-PA1/04/03-mTFP1 | [4] |
| PAO1:eYFP | pUC18-miniTn7T2-PA1/04/03-eYFP | [4] |
| PAO1:tdTomato | pUC18-miniTn7T2.1-Gm- GW::PA1/04/03-tdTomato | Plasmid provided by from Tseng Lab |
| *ΔkdpFABCDE* | PAO1 with of markerless, in frame deletion *kdpFABCDE* | This study |
| *ΔkdpFABCDE:*GFP | PAO1 with of markerless, in frame deletion *kdpFABCDE,* constitutively expressing GFP | This study |
| *∆kdpFABCDE,* attTn7::GFP, pJM220-kdpFA, pBBR5pemIK-kdpBC, pJM253-kdpDE | PAO1 with of markerless, in frame deletion *kdpFABCDE* complemented with *kdpFABCDE* on three plasmids. | This study |
| PAO1*ΔpilA* | PAO1 with markerless, in frame deletion of *pilA* | [5] |
| PAO1*ΔPA5518* | PAO1 with of markerless, in frame deletion of *PA5518* | This study |
| mPAO1 | Parent PAO1 strain of PAO1 transposon library | [6, 7] |
| mPAO1*kdpA::Tn* | Transposon mutant; PW3910;  Tn ISlacZ/hah inserted into PA1633 (kdpA) | [6, 7] |
| mPAO1*kdpC::Tn* | Transposon mutant; PW3913;  Tn ISlacZ/hah inserted into PA1635 (kdpC) | [6, 7] |
| mPAO1*kdpD::Tn* | Transposon mutant; PW3914;  Tn ISlacZ/hah inserted into PA1636 (kdpD) | [6, 7] |
| mPAO1*trkH::Tn* | Transposon mutant; PW6372;  Tn ISlacZ/hah inserted into PA3210 (trkH) | [6, 7] |
| mPAO1*trkA::Tn* | Transposon mutant; PW1016;  Tn ISlacZ/hah inserted into PA0016 (trkA) | [6, 7] |
| mPAO1*kup::Tn* | Transposon mutant; PW2666;  Tn ISlacZ/hah inserted into PA0917 (kup) | [6, 7] |
| mPAO1*PA1496::Tn* | Transposon mutant; PW3697;  Tn ISlacZ/hah inserted into PA1496 | [6, 7] |
| mPAO1*kefB::Tn* | Transposon mutant; PW3182;  Tn ISlacZ/hah inserted into PA1207 (kefB) | [6, 7] |

**REFERENCE**

1. Moreau-Marquis S, Bomberger JM, Anderson GG, Swiatecka-Urban A, Ye S, O'Toole GA, et al. The DeltaF508-CFTR mutation results in increased biofilm formation by Pseudomonas aeruginosa by increasing iron availability. Am J Physiol Lung Cell Mol Physiol. 2008;295(1):L25-37. Epub 2008/03/25. doi: 10.1152/ajplung.00391.2007. PubMed PMID: 18359885; PubMed Central PMCID: PMCPMC2494796.

2. Moreau-Marquis S, O'Toole GA, Stanton BA. Tobramycin and FDA-approved iron chelators eliminate Pseudomonas aeruginosa biofilms on cystic fibrosis cells. Am J Respir Cell Mol Biol. 2009;41(3):305-13. Epub 2009/01/27. doi: 10.1165/rcmb.2008-0299OC. PubMed PMID: 19168700; PubMed Central PMCID: PMCPMC2742750.

3. Zemke AC, Shiva S, Burns JL, Moskowitz SM, Pilewski JM, Gladwin MT, et al. Nitrite modulates bacterial antibiotic susceptibility and biofilm formation in association with airway epithelial cells. Free Radic Biol Med. 2014;77:307-16. Epub 2014/09/18. doi: 10.1016/j.freeradbiomed.2014.08.011. PubMed PMID: 25229185; PubMed Central PMCID: PMCPMC4278422.

4. Zhao K, Tseng BS, Beckerman B, Jin F, Gibiansky ML, Harrison JJ, et al. Psl trails guide exploration and microcolony formation in Pseudomonas aeruginosa biofilms. Nature. 2013;497(7449):388-91. Epub 2013/05/10. doi: 10.1038/nature12155. PubMed PMID: 23657259; PubMed Central PMCID: PMCPMC4109411.

5. Armbruster CR, Lee CK, Parker-Gilham J, de Anda J, Xia A, Zhao K, et al. Heterogeneity in surface sensing suggests a division of labor in Pseudomonas aeruginosa populations. Elife. 2019;8. Epub 2019/06/11. doi: 10.7554/eLife.45084. PubMed PMID: 31180327; PubMed Central PMCID: PMCPMC6615863.

6. Held K, Ramage E, Jacobs M, Gallagher L, Manoil C. Sequence-verified two-allele transposon mutant library for Pseudomonas aeruginosa PAO1. J Bacteriol. 2012;194(23):6387-9. Epub 2012/09/18. doi: 10.1128/JB.01479-12. PubMed PMID: 22984262; PubMed Central PMCID: PMCPMC3497512.

7. Jacobs MA, Alwood A, Thaipisuttikul I, Spencer D, Haugen E, Ernst S, et al. Comprehensive transposon mutant library of Pseudomonas aeruginosa. Proc Natl Acad Sci U S A. 2003;100(24):14339-44. Epub 2003/11/18. doi: 10.1073/pnas.2036282100. PubMed PMID: 14617778; PubMed Central PMCID: PMCPMC283593.
