# Supplementary material for: Pseudomonas aeruginosa senses and responds to epithelial potassium flux via Kdp operon to promote biofilm
Source: PLoS Pathog. 2024 May 31;20(5):e1011453. doi: 10.1371/journal.ppat.1011453 (PMC11168685; doi:10.1371/journal.ppat.1011453)
Supplement: S2 Table — Underlined region in primers represent attB sequence added to primer. (DOCX) [file ppat.1011453.s007.docx]

**S2 Table**

| **Primer** | **Sequence (5’ → 3’)^a^** |
| --- | --- |
| KdpAll Up-F | GGGGACAAGTTTGTACAAAAAAGCAGGCTACCTGCTGGGCACCAACAACG |
| KdpAll Up-R | GCGCAGGCGATAACCGACGACGAACAGTCCCGTGGC |
| KdpAll Down-F | GTCGGTTATCGCCTGCGC |
| KdpAll Down-R | GGGGACCACTTTGTACAAGAAAGCTGGGTAGCGGTTTCAGAAGATCGACC |
| KdpAll Seq-F | GAGCATCGCCTTCGAGCT |
| KdpAll Seq-R | GGCGACGCGATACAAGAC |
| PA5518 Up-F | GGGGACAAGTTTGTACAAAAAAGCAGGCTACCCACCATCACCGCCATCA |
| PA5518 Up-R | GAGTCCTGATGCACCATACGCGGACTCGACCGTGACGA |
| PA5518 Down-F | TCGTCACGGTCGAGTCCGCGTATGGTGCATCAGGACTC |
| PA5518 Down-R | GGGGACCACTTTGTACAAGAAAGCTGGGTAGGTCGCTACGTGGTGACC |
| PA5518 Seq-F | GGTTCGTTTCGACGCAGTG |
| PA5518 Seq-R | GCGAGATTGTTCAGCGCCTG |
